# *Paederia scandens* extract alleviates obesity via modulating the gut microbiota and serum metabolome disorder

**DOI:** 10.3389/fmicb.2025.1554537

**Published:** 2025-04-23

**Authors:** Yuanyuan Yang, Jinglei Si, Jiayuan Mo, Jin Li, Bin Pan, Yi Pan, Lihe Jiang, Decai Wang, Xueping Feng

**Affiliations:** ^1^School of Basic Medicine, Youjiang Medical University for Nationalities, Baise, China; ^2^College of Animal Science & Technology, Guangxi University, Nanning, China; ^3^College of Animal Science, Anhui Science and Technology University, Chuzhou, China; ^4^Laboratory Animal Center, Youjiang Medical University for Nationalities, Baise, China; ^5^Library, Youjiang Medical University for Nationalities, Baise, China

**Keywords:** *Paederia scandens*, obesity, high-fat induced, microbes, metabolites

## Abstract

Obesity is increasingly becoming a challenge with China’s economic development. There is an urgent need to identify more affordable methods to combat this condition. *Paederia scandens* (PS), a cost-effective herbal remedy widely used in China for treating inflammation and pain, shows potential in this regard. To investigate its anti-obesity mechanisms, we established a high-fat diet (HFD)-induced obesity model in mice. The obese mice subsequently received daily oral gavage of PS extract for 21 consecutive days. Upon the completion of the experiment, blood samples were collected to analyze lipid profiles, including total cholesterol (TC), triglycerides (TG), high-density lipoprotein (HDL-C), and low-density lipoprotein (LDL-C). Abdominal adipose tissue was subjected to hematoxylin–eosin (HE) staining for histological analysis, while fecal samples underwent 16S rRNA sequencing to assess gut microbiota composition. Our findings revealed that PS supplementation significantly reduced body weight, lipid metabolism biomarkers, and adipocyte size. PS treatment also restored gut microbial diversity, with 19 specific microbial taxa and 25 KEGG pathways identified as potential mediators of its anti-obesity effects. Notably, PS modulated key obesity-associated gut microbiota, including *Alistipes*, *Lachnoclostridium*, *Odoribacter*, *Prevotellaceae UCG-001*, *Rikenellaceae RC9-gut group*, and *norank_g Bacteroidales S24-7 group*. Serum metabolomics analysis implicated L-ascorbic acid, stevioside, allopurinol, and gingerol, along with amino acid and energy metabolism pathways, in the anti-obesity mechanism of PS. These results provide novel theoretical insights into the therapeutic potential of PS for obesity prevention and treatment.

## 1 Introduction

Metabolic diseases have emerged as a critical global health challenge (Wu Y. et al., 2024), with obesity representing a central contributor to this epidemic (Zhang et al., 2023). Recent data reveal that 50% of Chinese adults and 25% of children meet the criteria for obesity risk (Wang et al., 2021), a condition projected to incur global economic costs exceeding USD 4.32 trillion annually by 2035 (Mahase, 2023). This escalating crisis has intensified demands for innovative interventions targeting obesity-related mechanisms, including gut microbiota modulation and metabolic regulation (The Lancet Diabetes Endocrinology, 2021).

*Paederia scandens* (PS), a cost-effective botanical deeply rooted in Chinese traditional medicine, presents unique therapeutic potential (Wang et al., 2014), and is widely consumed as both a medicinal herb and a dietary component in Hainan Province (China). Phytochemical analyses have identified multiple bioactive constituents in PS, including 6’-O-E-feruloylmonotropein (Trung et al., 2023), iridoid glycosides (Xu et al., 2023), and paederosidic acid (Li et al., 2023). Experimental studies demonstrate its multifunctional properties: ameliorating non-alcoholic fatty liver disease through antioxidant enhancement in chickens (Wu et al., 2019), suppressing inflammatory pathways to alleviate diarrhea in mice (Ai et al., 2023), and restoring gut microbial balance in arthritis models (Xiao et al., 2018). These findings collectively position PS as a promising candidate for metabolic regulation, though its anti-obesity mechanisms remain unexplored.

Growing evidence implicates gut microbiota dysbiosis in obesity pathogenesis (Gomes et al., 2018). Specific microbial taxa exhibit distinct roles: *Megamonas rupellensis* promotes lipid absorption (Wu C. et al., 2024), *Fusimonas intestine* and *Lachnospiraceae* facilitate diet-induced obesity (Takeuchi et al., 2023), and *Bacteroidetes* demonstrates anti-obesity correlations (Ley et al., 2006). Therapeutic agents such as spermidine (Ma et al., 2020) and ginsenoside Rg1 (Liu et al., 2023) mitigate obesity via microbiota modulation, suggesting microbial targets for PS intervention. Concurrently, serum metabolomic studies reveal obesity-associated perturbations in glutamate (Liu et al., 2017) and branched-chain amino acids (Wahl et al., 2012), though PS’s metabolic regulatory effects remain uncharacterized.

To address these knowledge gaps, we established a high-fat diet-induced murine obesity model to systematically investigate PS’s anti-obesity efficacy. PS was orally administered to investigate its effects on body weight, biochemical indicators, and histopathological changes in obese mice. 16S rRNA and metabolomic sequencing were used to determine whether PS supplementation ameliorated fecal microbial dysregulation and serum metabolite disorders in the obesity model. Finally, we identified gut microbes and serum metabolites associated with the underlying protective mechanisms of PS against obesity. This multidisciplinary approach provides critical insights into PS’s therapeutic potential while advancing strategies for combating metabolic disorders.

## 2 Materials and methods

### 2.1 Animals and groups

In this study, specific pathogen-free (SPF) male C57BL/6 J mice (aged 5 weeks; body weight 19.97 ± 0.93 g) were obtained from RUIYE laboratories (Guangzhou, China; Production License for Experimental Animals: SCXK(YUE)2022–0063; Certificate No.44829700021758). A total of 22 mice were randomly assigned to 3 groups: normal control (NC, *n* = 6), obesity model (OB, *n* = 8), and PS extract treatment (PS, *n* = 8). All animals were housed at the Experimental Animal Center of Youjiang Medical University for Nationalities (Laboratory Animal Use License: SYXK(GUI)2022–0004) under controlled conditions: temperature 22 ± 2°C, relative humidity 55 ± 5%, and 12-h light/dark cycles. The NC group received a standard diet throughout the 13-week experiment, while the OB and PS groups were fed a high-fat diet (HFD) (4.73 kcal/g digestible energy, Charles River Co., Ltd., Jiaxing, China). From weeks 10 to 13, the NC and OB groups were administered normal saline (1 mL/kg/day), whereas the PS group received the PS extraction solution. All animal feeding, management, and experiment procedures strictly comply with the *Regulations on the Management of Experimental Animals* (China National Standard GB/T 35823–2018).

### 2.2 PS extract preparation

PS extract was prepared by boiling 200 g of dried PS raw material (Zexintang Pharmaceutical Co., Ltd., Bozhou, China) in 1000 mL of double-distilled water. The solution was concentrated to 100 mL under reduced pressure, yielding extract with a final PS concentration of 2 g/mL.

### 2.3 Physiological, biochemical indexes, and sample collection

The weekly body weight was recorded for all mice. Feces samples were collected at week 13. Following isoflurane anesthesia, blood and abdominal adipose tissue were harvested. Blood samples were centrifuged (3,000 rpm, 15 min, 4°C) to isolate serum. Serum total cholesterol (TC) (A111-1-1), triglycerides (TG) (A110-1-1), high-density lipoprotein cholesterol (HDL-C) (A112-1-1), and low-density lipoprotein cholesterol (LDL-C) (A113-1-1) were quantified using ELISA kits (Jiancheng Bioengineering Institute, Nanjing, China). Abdominal fat was fixed in 4% paraformaldehyde and embedded in paraffin blocks (4 μm thickness). Feces samples were immediately snap-frozen in liquid nitrogen and stored at −80°C for subsequent analysis.

### 2.4 16s rRNA amplicon sequencing

Fecal microbial genomic DNA was extracted using the MagPure Soil DNA LQ Kit (Magen, Cat. No. D6356-02; Guangzhou, China), following the manufacturer’s protocol. DNA quality was verified by NanoDrop 2000 spectrophotometry and 1.5% agarose gel electrophoresis. The V3–V4 region of 16S rRNA was amplified via PCR using barcoded primers 343F (5’-TACGGRAGGCAGCAG-3′) and 798R (5’-AGGGTATCTAATCCT-3′). Libraries were constructed and sequenced on the Illumina MiSeq 6000 platform (OE Biotech, Shanghai, China). The analysis pipeline for 16S rRNA was completed according to a previous study (Mo et al., 2022). Briefly, the raw sequences were analyzed using EasyAmplicon v1.0 with the minimum unique size in the dereplication, and the sintax_cutoff in the removal of plastids and non-bacteria was 12 and 0.1, respectively. After quality control, all the samples were subsampled to 57735 sequences per sample and aligned using the silva_16s_v123 database. Beta diversity was calculated using unweighted UniFrac distances and visualized via principal coordinate analysis (PCoA). The LDA score ≥4 was defined as significant for differential microbes using linear discriminant analysis effect size (Lefse) software. The different KEGG pathways were identified by STAMP software with Bonferroni corrections (*p* < 0.05).

### 2.5 Serum untargeted metabolomics

After thawing on ice, each serum sample (80 μL) was transferred to 2 mL Eppendorf (EP) tubes containing 400 μL of protein precipitator solvent (ACN:methanol = 1:2, V/V) spiked with internal standards. Samples were sonicated (10 min) and allowed to equilibrate at −40°C overnight. Following centrifugation (12,000 rpm, 20 min, 4°C), 150 μL of supernatant was transferred to sample vials for liquid chromatography-mass spectrometry (LC–MS) analysis at Oebiotech Company (Shanghai, China). LC–MS analysis was performed using an ACQUITY UPLC HSS T3 column (100 mm × 2.1 mm, 1.8 μm, Waters, Milford, MA, USA). Mass spectrometry parameters for positive/negative ion modes are detailed in Table 1. Chromatographic gradients (mobile phase composition) and instrumental parameter profiles are provided in Tables 2, 3, respectively.

**Table 1:** The MS conditions of position and negative ion model.

Item	ESI+	ESI−
Spray Voltage (V)	3,800	−3,200
Capillary Temperature (°C)	320	320
Aux gas heater temperature (°C)	350	350
Sheath Gas Flow Rate (Arb)	35	35
Aux gas flow rate (Arb)	8	8
S-lens RF level	50	50
Mass range (m/z)	70–1,050	70–1,050
Full ms resolution	60,000	60,000
MS/MS resolution	15,000	15,000
NCE/stepped NCE	10, 20, 40	10, 20, 40

**Table 2:** Instrument operation program of LC–MS analysis.

Item	Parameter
Column temperature	45°C
Flow rate	0.35 mL/min
Sample injection volume	4 μL
Mobile phase A	Water plus 0.1% formic acid
Mobile phase B	Acetonitrile

**Table 3:** Changes of solvents in gradient elution of LC–MS analysis.

Time (min)	A (%)	B (%)
0.0	95.0	5.0
2.0	95.0	5.0
4.0	70.0	30.0
8.0	50.0	50.0
10.0	20.0	80.0
14.0	0.0	100.0
15.0	0.0	100.0
15.1	95.0	5.0
16.0	95.0	5.0

Raw LC–MS data were processed through peak filtration, peak extraction, alignment, and retention time correction using XCMS software (v4.5.1). Metabolite annotation was performed by querying the Human Metabolome Database (HMDB), LipidMaps (v2.3), METLIN, and LuMet-Animal 3.0 databases. Then the principal component analysis (PCA), supervised orthogonal partial least squares discriminant analysis (OPLS-DA), permutation test (200 permutations), and Kyoto Encyclopedia of Genes and Genomes (KEGG) analysis were conducted following established protocols (Mo et al., 2021). Metabolites with specific variable importance in projection (VIP) scores (VIP > 2), fold changes (FC) (FC > 4 or FC < 0.25), and *p*-values (*p* < 0.001) were defined as significantly differential metabolites. Time-series clustering of metabolite profiles was performed using STEM software (see Table 4).

**Table 4:** The restored KEGG pathway by the PS added.

Pathway	NC	OB	PS
Glycosphingolipid biosynthesis - ganglio series	74,172	71,903	86,032
beta-Lactam resistance	14,928	19,423	17,909
Biotin metabolism	79,087	106,166	93,284
Cellular antigens	32,393	44,048	38,634
Cell division	47,207	64,626	55,543
Lipoic acid metabolism	31,208	43,105	37,163
Peroxisome	98,895	137,458	116,658
Folate biosynthesis	181,353	267,903	216,888
Lipopolysaccharide biosynthesis	174,882	260,702	214,077
Penicillin and cephalosporin biosynthesis	14,955	22,689	18,350
Nicotinate and nicotinamide metabolism	173,909	270,089	201,843
Carbon fixation pathways in prokaryotes	431,821	679,188	518,126
Phenylpropanoid biosynthesis	73,053	114,951	101,043
Vitamin B6 metabolism	87,017	137,776	103,411
Chaperones and folding catalysts	417,051	666,043	497,665
Phosphatidylinositol signaling system	35,155	57,206	43,203
Cyanoamino acid metabolism	126,099	209,387	165,213
Glycine, serine and threonine metabolism	332,589	560,344	393,471
Starch and sucrose metabolism	322,937	591,088	421,056
Glycerolipid metabolism	116,271	239,487	137,455
Porphyrin and chlorophyll metabolism	139,159	429,877	199,871
Steroid hormone biosynthesis	2,822	10,111	8,057
Synthesis and degradation of ketone bodies	3,248	16,536	3,296
Prion diseases	965	6,681	1,206
Atrazine degradation	2,389	21,295	3,032

## 3 Results

### 3.1 Addition of PS reduced obesity related indices

**Figure 1:**
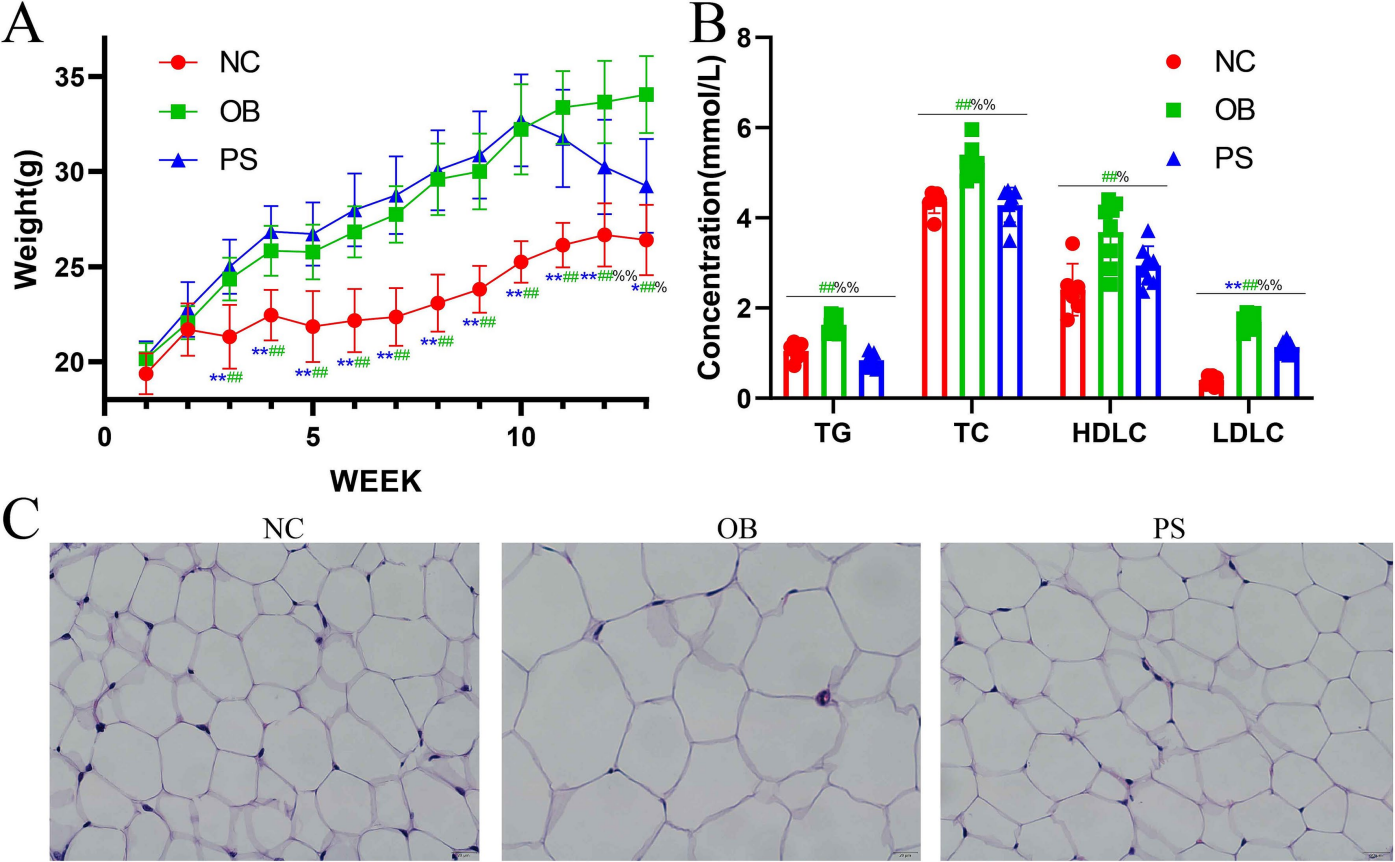
Indices and hematoxylin–eosin (HE)-stained sections from obesity. **(A)** body weight; **(B)** TG, TC, HDL-C, and LDL-C concentrations in blood; **(C)** HE-stained section of abdominal fat in three groups. Note: The ** was the extremely significant difference between the NC group and the PS group (*p* < 0.01); the ## was the extremely significant difference between the NC group and the OB group (*p* < 0.01); the %% was the extremely significant difference between the OB group and the PS group (*p* < 0.01); the * was the significant difference between the NC group and the PS group (*p* < 0.05); the # was the significant difference between the NC group and the OB group (*p* < 0.05); and the % was the significant difference between the OB group and the PS group (*p* < 0.05).

During the first and second weeks, the mean body weight of mice in the NC, OB, and PS groups showed no significant difference (*p* > 0.05) (Figure 1A). Notably, from weeks 3 to 12, HFD-fed mice (OB and PS groups) showed significantly elevated body weights compared to the NC group (*p* < 0.01) (Figure 1A). By week 13, the body weight in the PS group was significantly higher than in the NC group (*p* < 0.05; Figure 1A). Intriguingly, no significant differences were observed between the OB and PS groups from weeks 1 to 10 (*p* > 0.05; Figure 1A). Following PS administration (weeks 11–13), the PS group exhibited progressive weight reduction, culminating in significantly lower body weight versus the OB group at week 13 (*p* < 0.01; Figure 1A). These findings suggest that PS supplementation ameliorates HFD-induced weight gain. Serum TC, TG, HDL-C, and LDL-C in the OB group were markedly elevated compared to the NC and PS groups (*p* < 0.05; Figure 1B). Similarly, LDL-C levels in the NC group were significantly suppressed relative to the PS group (*p* < 0.05; Figure 1B). Histological analysis of inguinal adipose tissue revealed that the size of fat cells in the OB group was larger than those in the NC and PS groups (*p* > 0.05; Figure 1C). Critically, adipocyte sizes in the PS group did not statistically match those in the NC group (*p* > 0.05), indicating PS-mediated normalization of adipocyte morphology (Figure 1C). Thus, our results show that HFD exacerbates obesity-related metabolic indices and adipocyte hypertrophy, and PS intervention attenuates HFD-induced dyslipidemia and adipose remodeling.

### 3.2 Addition of PS changes the microbe diversity

**Figure 2:**
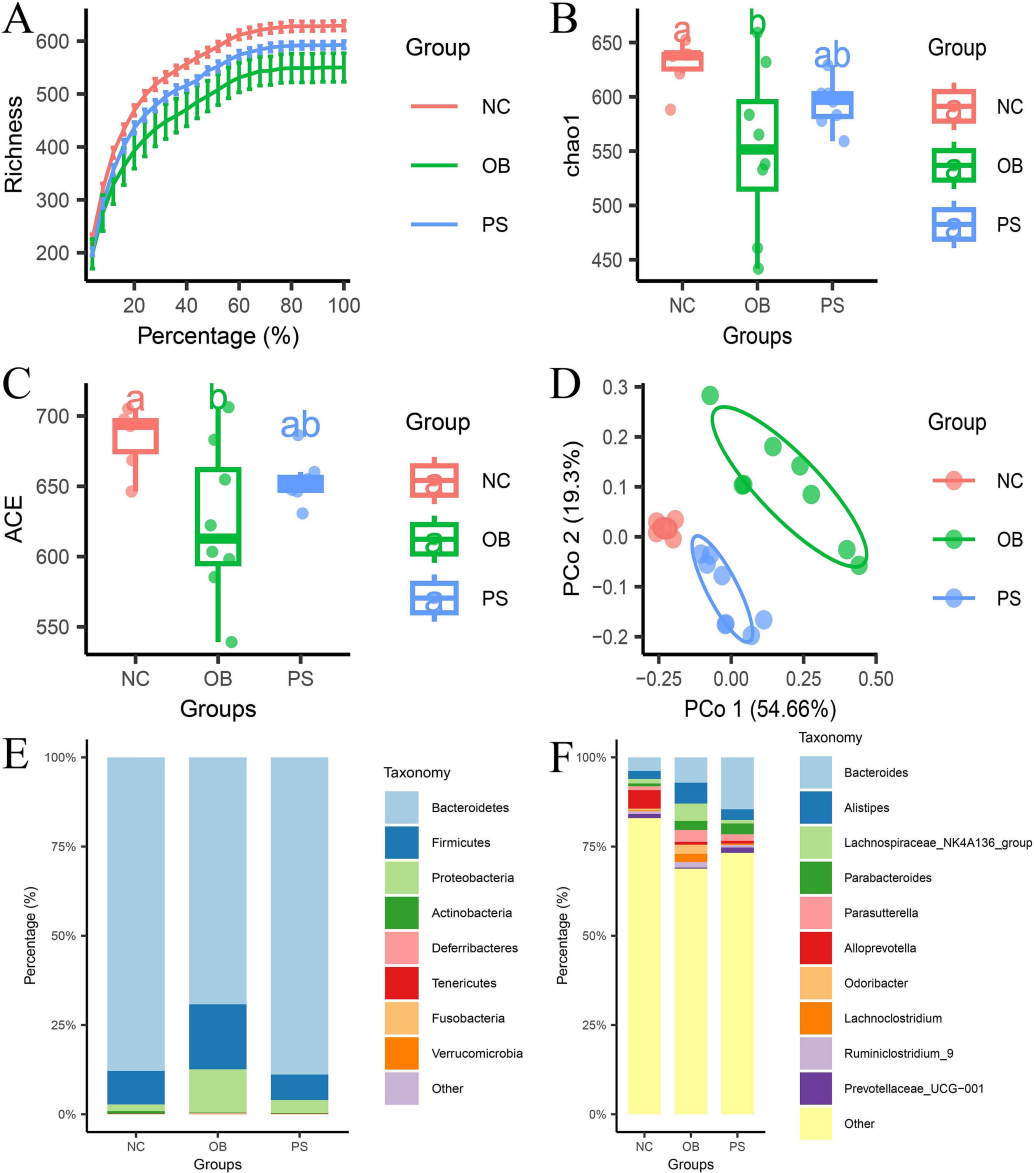
Microbial composition analysis. **(A)** the rarefaction curve in three groups; **(B)** the Chao1 index in three groups; **(C)** the ACE index in three groups; **(D)** *β*-diversity in the three groups; **(E)** the microbial composition at the phylum level; and F: the microbial composition in the genus level.

The difference in fecal microbiota among the NC, OB, and PS groups was identified using 16S rRNA sequencing. After quality control and dereplication, a total of 1,397,098 reads were obtained, with the number of available sequences ranging from 78,153 to 81,920. The rarefaction curve analysis showed that the data met the requirements for bioinformatics (Figure 2A). A total of 794 amplicon sequence variants (ASVs) were identified, with 169 ASVs (21.3%) shared by all samples and 316 ASVs (39.8%) present in over 90% of the samples. After normalization to 57,735 sequences per sample, both the ACE and Chao1 indices in the OB group were significantly lower than those in the NC group (*p* < 0.05; Figures 2B,C). However, no significant differences were observed between the PS and NC groups in these indices (*p* > 0.05; Figures 2B,C). *β*-diversity analysis revealed structure differences in microbiota composition among the three groups (Figure 2D). Principal coordinates (PCo) 1 and 2 accounted for 54.66 and 19.3% of the total variation, respectively (Figure 2D). Notably, the NC group exhibited closer clustering with the PS group than with the OB group (Figure 2D).

### 3.3 Addition of PS changes the microbe composition

The relative abundances of bacteria are shown at the phylum level (Figure 2E) and the genus level (Figure 2F). A total of 8 phyla, 17 classes, 21 orders, 38 families, and 92 genera were identified in this study. The major bacteria in the phyla were Bacteroidetes, Firmicutes, and Proteobacteria, accounting for over 95.7% of the total in each sample. The relative abundance of Bacteroidetes in the NC, OB, and PS groups was 87.87, 69.22, and 88.89%, respectively. The relative abundance of Firmicutes in the NC, OB, and PS groups was 9.41, 18.23, and 7.14%, respectively. The relative abundance of Proteobacteria in the NC, OB, and PS groups was 1.84, 12.07, and 3.64%, respectively. These results showed that the microbial composition at the phylum level in the PS group was closer to that of the NC group than to that of the OB group. The main bacterial genera in the NC group at the genus level were *Alloprevotella*, *Bacteroides*, *Alistipes*, *Ruminococcaceae UCG-014*, and the *Lachnospiraceae NK4A136 group*. The main bacterial genera in the OB group at the genus level were *Bacteroides*, *Alistipes*, *Lachnospiraceae NK4A136 group*, *Parasutterella*, *Odoribacter*, *Parabacteroides*, and *Lachnoclostridium.* The main bacterial genera in the PS group at the genus level were *Bacteroides*, *Parabacteroides*, *Alistipes*, *Parasutterella*, and *Rikenellaceae RC9-gut group.* These results indicate that the addition of PS changed the dominant microbial composition in feces, and these changes may be related to the protective mechanism of PS against HFD-induced obesity in mice.

### 3.4 Difference in microbes and pathways between the NC and OB groups

**Figure 3:**
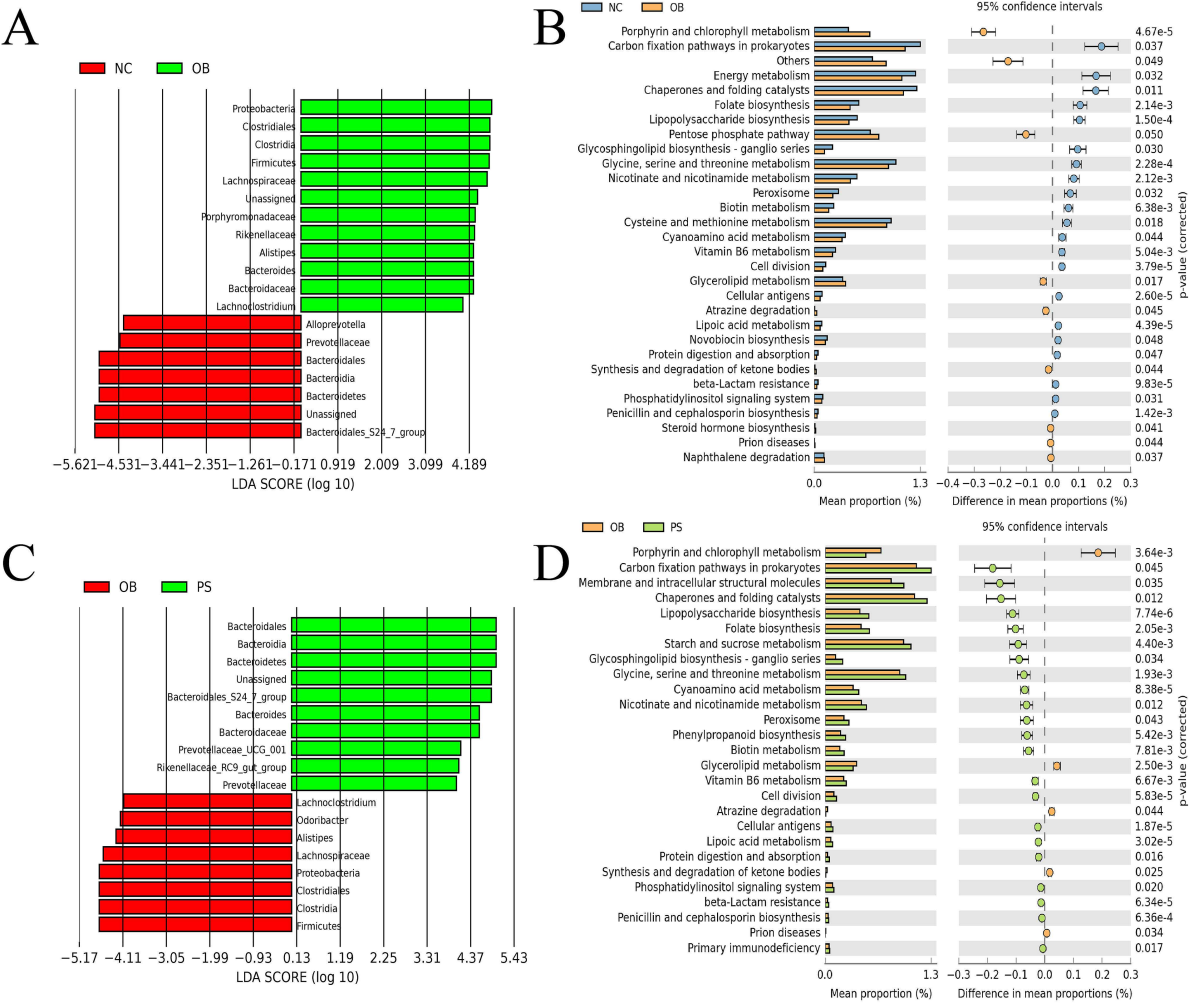
Difference in microbes and pathways. **(A)** difference in microbes between the NC and OB groups. **(B)** the difference in pathways between the NC and OB groups. **(C)** difference in microbes between the OB and PS groups. **(D)** difference in pathways between the OB and PS groups.

The relative abundances of 19 microbes showed significant differences between the NC and OB groups (*p* < 0.05), including *Alloprevotella*, *norank_g Bacteroidales S24-7 group*, Bacteroidales S24-7 group, Prevotellaceae, Bacteroidales, Bacteroidia, and Bacteroidetes, which were significantly higher in the NC group than in the OB group (Figure 3A). The relative abundance of *Alistipes*, *Bacteroides*, *Lachnoclostridium*, *norank_g Enterobacteriaceae*, Bacteroidaceae, Lachnospiraceae, Porphyromonadaceae, Rikenellaceae, Clostridiales, Clostridia, Firmicutes, and Proteobacteria was significantly lower in the NC group than in the OB group (Figure 3A). The KEGG pathway analysis revealed that 21 KEGG pathways in the NC group were significantly more abundant than those in the OB group, including glycosphingolipid biosynthesis - ganglio series, protein digestion and absorption, biotin metabolism, lipoic acid metabolism, lipopolysaccharide biosynthesis, folate biosynthesis, nicotinate and nicotinamide metabolism, carbon fixation pathways in prokaryotes, vitamin B6 metabolism, and glycine, serine and threonine metabolism pathways, among others (Figure 3B). Additionally, nine KEGG pathways in the NC group were significantly less abundant than those in the OB group, including glycerolipid metabolism, steroid hormone biosynthesis, and synthesis and degradation of ketone bodies pathways, among others (Figure 3B).

### 3.5 Difference in microbes and pathways between the OB and PS groups

The relative abundances of 18 microbes showed significant differences between the OB and PS groups (*p* < 0.05), including *Alistipes*, *Lachnoclostridium*, *Odoribacter*, Lachnospiraceae, Clostridiales, Clostridia, Firmicutes, and Proteobacteria, which were significantly higher in the OB group than in the PS group (Figure 3C). The relative abundance of *Bacteroides, Prevotellaceae UCG-001, Rikenellaceae RC9-gut group, norank_g Bacteroidales S24-7 group*, Bacteroidaceae, Bacteroidales S24-7 group, Prevotellaceae, Bacteroidales, Bacteroidia, and Bacteroidetes was significantly lower in the OB group than in the PS group (Figure 3C). The KEGG pathway analysis revealed that five KEGG pathways in the OB group were significantly more abundant than those in the PS group, including synthesis and degradation of ketone bodies and glycerolipid metabolism pathways (Figure 3D). Additionally, 22 KEGG pathways in the OB group were significantly less abundant than those in the PS group, including glycine, serine, and threonine metabolism; starch and sucrose metabolism; nicotinate and nicotinamide metabolism; vitamin B6 metabolism; carbon fixation pathways in prokaryotes; folate biosynthesis; lipopolysaccharide biosynthesis; lipoic acid metabolism; biotin metabolism; glycosphingolipid biosynthesis—ganglio series; and protein digestion and absorption pathways, among others (Figure 3D).

### 3.6 Addition of PS restored the disorder of the feces microbe

**Figure 4:**
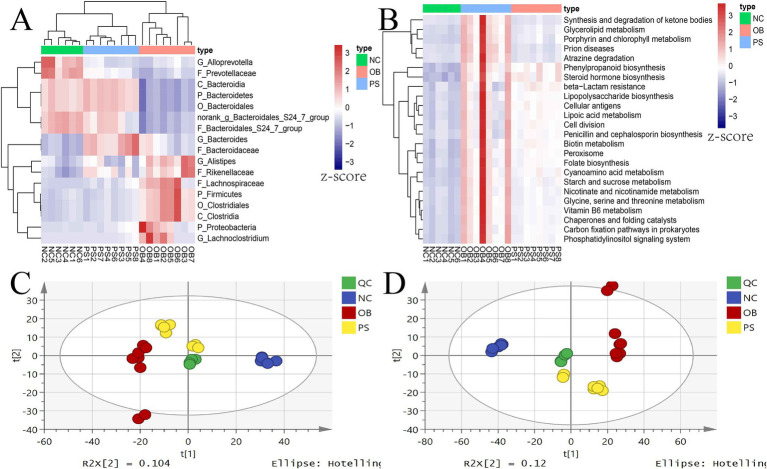
Microbial taxa and pathways related to PS anti-obesity and the PCA plot of metabolites. **(A)** the microbial taxa related to PS anti-obesity; **(B)** the microbial pathways related to PS anti-obesity; **(C)** the PCA plot in the positive ion model; and **(D)** the PCA plot in the negative ion model.

Based on microbial differences among the NC, OB, and PS groups, our study identified seven microbial taxa in the OB group with significantly higher relative abundance than those in both the NC and PS groups, including *Lachnoclostridium*, *Alistipes*, Proteobacteria, Firmicutes, Clostridiales, Lachnospiraceae, and Clostridia (*p* < 0.05). Meanwhile, the relative abundance of these taxa in the PS group was higher than that in the NC group, though not statistically significant (*p* > 0.05). Additionally, the relative abundance of Rikenellaceae was significantly higher in the OB and PS groups compared to the NC group (*p* < 0.05), with the OB group showing a non-significantly higher abundance than the PS group (*p* > 0.05). Thus, eight differential microbial taxa exhibited the highest abundance in the OB group, followed by the PS group, while the NC group showed the lowest levels (Figure 4A).

Three distinct microbial taxa in the OB group exhibited significantly lower relative abundances than those in both the NC and PS groups, including Bacteroidetes, Bacteroidales, and Bacteroidia (*p* < 0.05). Meanwhile, the relative abundance of these taxa in the PS group was higher than that in the NC group, though not statistically significant (*p* > 0.05). Thus, these three taxa showed the highest abundance in the PS group, followed by the NC group, and the lowest in the OB group. Additionally, three differential taxa in the NC group were significantly more abundant than those in the OB and PS groups (*p* < 0.05), with the PS group also showing significantly higher levels than the OB group (*p* < 0.05), including the Bacteroidales S24-7 group, Prevotellaceae, and *norank_g Bacteroidales S24-7 group*. Therefore, their abundance was highest in the NC group, intermediate in the PS group, and lowest in the OB group (Figure 4A). In summary, PS supplementation alleviated fecal microbial dysbiosis by reducing the abundance of *Lachnoclostridium*, *Alistipes*, Proteobacteria, Firmicutes, Clostridiales, Lachnospiraceae, Rikenellaceae, and Clostridia while enhancing the abundance of Bacteroidetes, Bacteroidales, Bacteroidia, Bacteroidales S24-7 group, Prevotellaceae, and *norank_g Bacteroidales S24-7 group.*

Meanwhile, the dysregulation of 25 significantly altered KEGG pathways was normalized by PS supplementation, such as the decrease of the glycosphingolipid biosynthesis—ganglio series pathway in the OB group was restored by PS intervention (Figure 4B). Additionally, the abnormally elevated levels of biotin metabolism, lipoic acid metabolism, folate biosynthesis, lipopolysaccharide biosynthesis, nicotinate and nicotinamide metabolism, carbon fixation pathways in prokaryotes, vitamin B6 metabolism, glycine, serine and threonine metabolism, starch and sucrose metabolism, glycerolipid metabolism, steroid hormone biosynthesis, and synthesis and degradation of ketone body pathways in the OB group were also restored by PS (Figure 4B). These findings suggest that PS may counteract obesity induced by HFDs by modulating these 25 microbial KEGG pathways.

### 3.7 Addition of PS changes the serum metabolites profiles

A total of 3,084 metabolites were identified in the serum metabolome, with 1,413 and 1,671 metabolites in the positive and negative ion modes, respectively (Supplementary Table S1). PCA results indicated that the model interpretation rates of X (R^2^X) for the positive and negative ion modes were 0.456 and 0.564, respectively. In both PCA score plots, the four quality control (QC) samples clustered tightly, confirming data reproducibility (Figures 4C,D). Notably, the PS group was positioned between the NC and OB groups in both the positive and negative ion modes.

### 3.8 Difference in metabolites and pathways between the NC and OB groups

**Figure 5:**
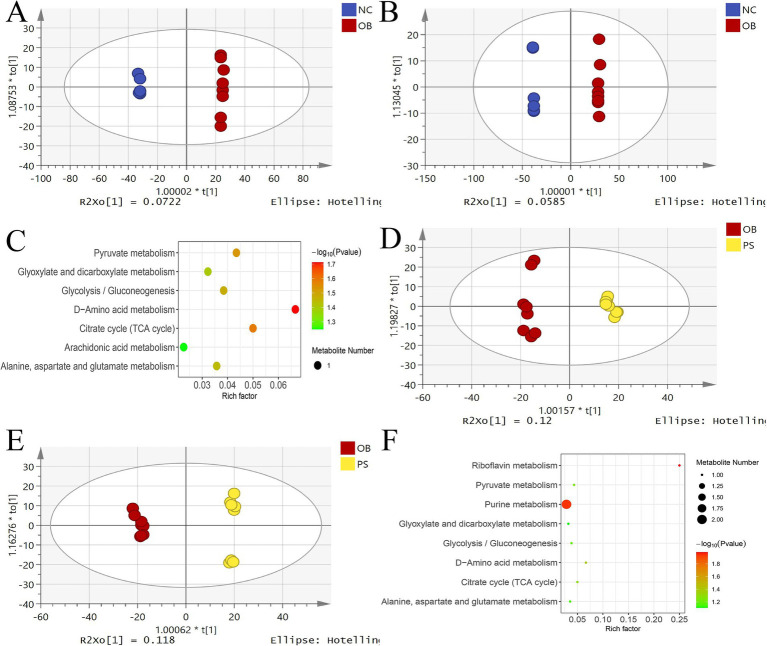
OPLS-DA analysis in different groups. **(A)** the OPLS-DA score plot in positive between the NC and OB groups; **(B)** the OPLS-DA score plot in negative between the NC and OB groups; **(C)** the KEGG plot of significantly different metabolites between the NC and OB groups; **(D)** the OPLS-DA score plot in positive between the PS and OB groups; **(E)** the OPLS-DA score plot in negative between the PS and OB groups; and **(F)** the KEGG plot of significantly different metabolites between the PS and OB groups.

OPLS-DA analysis revealed a clear separation between the NC and OB groups in both ion modes (Figures 5A,B). The model interpretation rates of Y (R^2^Y) in the positive and negative ion modes were 0.662 and 0.761, respectively. The prediction ability (Q^2^) in the positive and negative ion modes was over 0.99 in both modes. Permutation test intercepts for both modes were below −0.45, confirming model validity. Among 136 significantly differential metabolites, 56 were upregulated and 80 were downregulated in the NC group compared to the OB group (Supplementary Table S2). MetaboAnalyst 4.0 analysis identified seven enriched metabolic pathways, including D-amino acid metabolism, citrate cycle (TCA cycle), pyruvate metabolism, glycolysis/gluconeogenesis, alanine, aspartate and glutamate metabolism, glyoxylate and dicarboxylate metabolism, and the arachidonic acid metabolism pathway (Figure 5C).

### 3.9 Difference in metabolites and pathways between the OB and PS groups

OPLS-DA analysis revealed a clear separation between the OB and PS groups in both ion modes (Figures 5D,E). The R^2^Y values for the positive and negative ion modes were 0.437 and 0.488, respectively. The Q^2^ in both the positive and negative ion modes was over 0.93 in both modes. Permutation test intercepts for both modes were below −0.28, confirming model reliability. Among 60 significantly differential metabolites, 6 were upregulated and 54 downregulated in the OB group compared to the PS group (Supplementary Table S3). MetaboAnalyst 4.0 analyzes eight enriched metabolic pathways, including riboflavin metabolism, purine metabolism, D-amino acid metabolism, citrate cycle (TCA cycle), pyruvate metabolism, glycolysis/gluconeogenesis, alanine, aspartate and glutamate metabolism, and glyoxylate and dicarboxylate metabolism pathway (Figure 5F).

### 3.10 Addition of PS restored the disorder of serum metabolites

**Figure 6:**
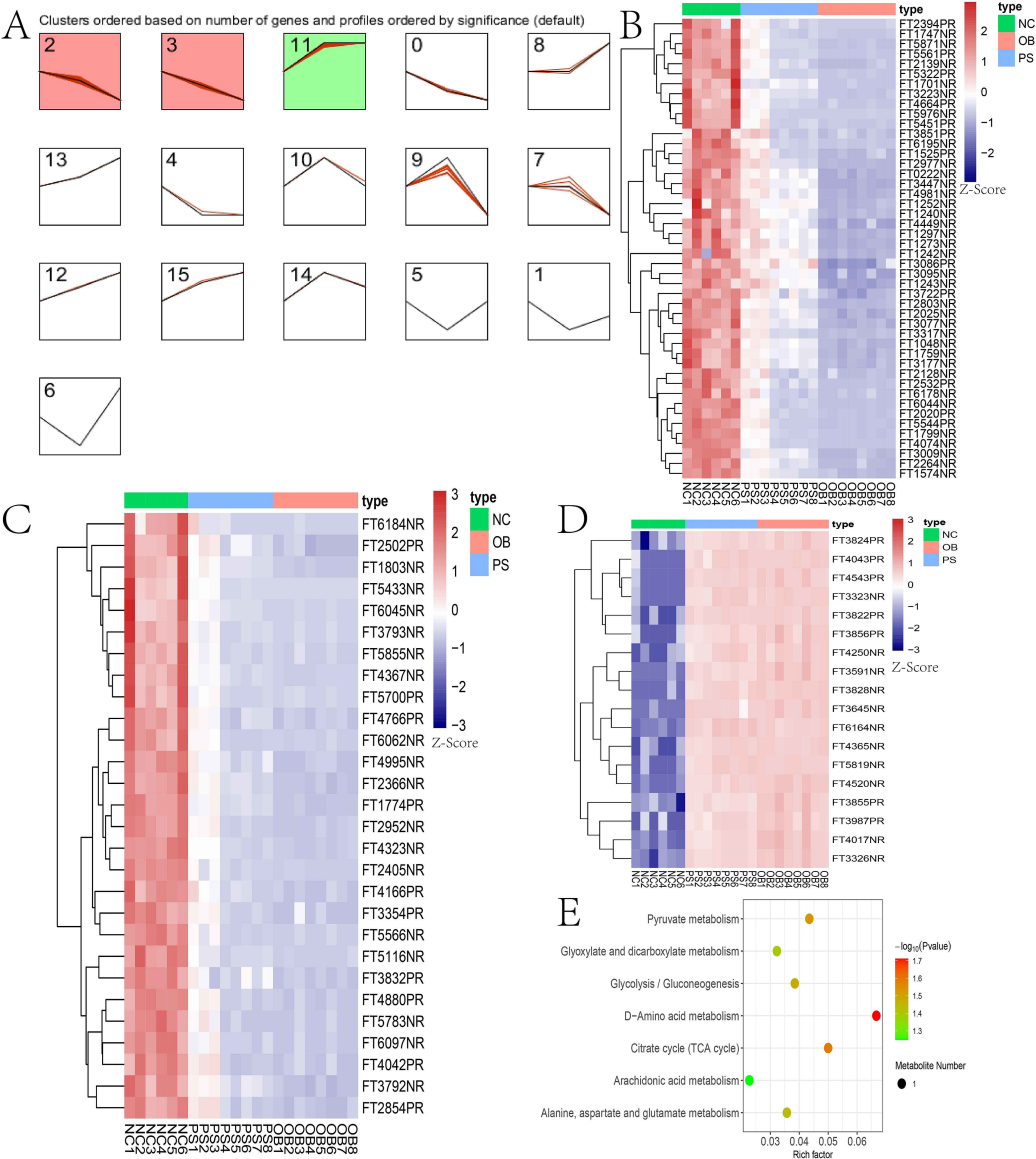
Hierarchical cluster analysis results. **(A)** the hierarchical cluster of 189 metabolites; **(B)** the metabolites in cluster 2; **(C)** the metabolites in cluster 3; **(D)** the metabolites in cluster 11; **(E)** the KEGG plot of clusters 2 and 3.

Hierarchical cluster analysis of all significant differential metabolites was performed using the Stem software. From the NC to PS and then OB groups, 189 significant difference metabolites were stratified into three distinct clusters (Figure 6A). Clusters 2, 3, and 11 contained 46, 28, and 18 metabolites, respectively (Supplementary Table S4; Figures 6B–D). Notably, metabolite concentration in clusters 2 and 3 of the PS group was intermediate between the NC and OB groups, suggesting that these 74 metabolites (cluster 2 + 3) may be associated with PS-mediated alleviation of HFD-induced obesity in mice. These metabolites were enriched in seven metabolic pathways, including D-amino acid metabolism, citrate cycle (TCA cycle), pyruvate metabolism, glycolysis/gluconeogenesis, alanine, aspartate and glutamate metabolism, glyoxylate and dicarboxylate metabolism, and the arachidonic acid metabolism pathway (Figure 6E).

## 4 Discussion

To investigate the protective mechanisms of PS against obesity, we established an HFD-induced obesity model in mice and administered PS via oral gavage to obese subjects. Our study demonstrated that HFD-fed mice exhibited significantly higher body weight than those on a standard diet, consistent with previous findings (Le Roy et al., 2022). Following PS intervention starting at week 10, the body weight of the PS group became lower than the OB group, with statistically significant differences emerging by week 13. These results indicate that PS mitigates high-fat diet-induced weight gain in mice. Similar to the effects of semaglutide (Ren et al., 2022), PS treatment significantly reduced serum lipid levels, including TC, TG, HDL-C, and LDL-C. Notably, TC, TG, and HDL-C levels in the PS group returned to normal ranges compared to the OB group. Additionally, the adipocyte size in the PS group was markedly smaller than in the OB group. Thus, our results showed that the oral administration of PS restored body weight, normalized blood lipid indices, and reduced adipocyte hypertrophy in obese mice. These results confirm that oral PS supplementation is an effective strategy for alleviating obesity in murine models.

Our microbial analysis revealed that HFD decreased *α*-diversity in mice, while PS intervention restored it to baseline levels. *β*-diversity metrics demonstrated closer clustering between the NC and PS groups compared to the OB group, indicating PS-mediated restoration of gut microbiota composition in obesity. Differential abundance analysis identified seven microbes that were decreased, and 12 microbes were enhanced in the OB group compared to the NC group. These findings align with prior studies of HFD-induced depletion of *Alloprevotella* and Prevotellaceae, alongside enrichment of *Alistipes* (Kong et al., 2019). Wei et al. (2020) indicated that the HFD decreased the abundance of the Bacteroidales S24-7 group and Bacteroidetes and increased the abundance of Firmicutes and Proteobacteria. Gong et al. (2022) reported that the abundance of *Lachnoclostridium* in the obesity group was enhanced. Duan et al. (2024) found that the abundance of Lachnospiraceae and *Bacteroides* in HFD mice was enhanced. Yang et al. (2021) evidenced that the abundance of Porphyromonadaceae in HFD mice was improved, and Daniel et al. (2014) declared that the abundance of Rikenellaceae in HFD mice was enhanced. Notably, PS treatment reversed 14 of 18 OB-associated microbial dysregulations, including eight microbes [*Alistipes* (Kong et al., 2019)*, Lachnoclostridium* (Gong et al., 2022), Lachnospiraceae (Duan et al., 2024), Rikenellaceae (Daniel et al., 2014), Clostridiales, Clostridia, Firmicutes, and Proteobacteria] that were decreased by the PS addition, and six microbes (*norank_g Bacteroidales S24-7 group* (Wei et al., 2020), Bacteroidales S24-7 group (Wei et al., 2020), Prevotellaceae (Kong et al., 2019), Bacteroidales, Bacteroidia, and Bacteroidetes) that were increased by the PS addition. Meanwhile, the abundance of *Odoribacter*, *Prevotellaceae UCG-001*, and *Rikenellaceae RC9-gut group* in the PS group was significantly higher than those in the OB group. *Lachnoclostridium* (Dicks, 2024) and *Alistipes* (Chanda and De, 2024) were the short-chain fatty acid (SCFA) producers, and the SCFAs can affect adipose tissue metabolism, lipid oxidation capacity, *β* cell function, and insulin secretion via the SCFA-sensing G protein-coupled receptors (GPCRs), SCFA transporter (SLC16A and SLC5A), and other SCFA receptor-independent pathways (You et al., 2022). The higher abundances of *Lachnoclostridium* and *Alistipes* in obesity samples in our study and previous studies may be related to the negative feedback mechanism of the body. The *norank_g Bacteroidales S24-7 group* was a butyrate-producing bacterium (Fang et al., 2020), and the butyrate can resist inflammation and obesity (Liu et al., 2018) through GPCR signaling pathways (Saban Güler et al., 2025). The relationship between the *Prevotellaceae UCG-001* and obesity in the body (Han et al., 2024) may be related to the butyrate because the butyrate can enhance the abundance of *Prevotellaceae UCG-001* in the gut (Wang et al., 2024). As a potentially obesity-related biomarker (Liu et al., 2022; Vallianou et al., 2023), the *Odoribacter* improves glucose control and inflammatory profile in obese mice by depleting circulating succinate (Huber-Ruano et al., 2022). A previous study showed that the *Rikenellaceae RC9-gut group* was related to HFD-induced obesity (Fu et al., 2022), and the *Rikenellaceae RC9-gut group* was positively correlated with HFD-induced “harmful indicators” and negatively correlated with “beneficial indicators” (Gao et al., 2020). Thus, PS might alleviate obesity in mice by restoring the abundance at the genus levels of *Alistipes, Lachnoclostridium, Odoribacter*, *Prevotellaceae UCG-001*, *Rikenellaceae RC9-gut group*, and *norank_g Bacteroidales S24-7 group*.

Our metabolomic profiling revealed that the PS group clustered intermediately between the NC and OB groups in both positive and negative ion modes, which was the same as the β-diversity results in the microbe analysis. This spatial distribution suggests the partial restoration of serum metabolite profiles in HFD-induced obese mice by PS intervention. Differential metabolite analysis identified 136 serum metabolites significantly altered by HFD, including cholestanetriol, gingerol, isovaleric acid, and stevioside. Zhu et al. (2021) illustrated that the HFD enhanced the serum cholestanetriol in mice, which was consistent with our study. Same as our study, isovaleric acid was an SCFA in the body and regulated obesity by decreasing lipogenesis (Heimann et al., 2016). Saravanan et al. (2014) reported that the gingerol could decrease the glucose level, body weight, leptin, insulin, amylase, lipase plasma, and tissue lipids in obesity mice. Brahma Naidu et al. (2016) proposed that it might modulate the lipid metabolism through decreasing activity in lipogenesis as well as increasing fatty-acid oxidation, which is contrary to our study. Wang et al. (2012) found that the stevioside may ameliorate insulin resistance in HFD mice by attenuating adipose tissue inflammation and inhibiting the NF-κB pathway. These results may be due to the negative feedback mechanism of the body. The KEGG analysis showed that two amino acid metabolism and three energy metabolism-related pathways were enriched in the different metabolites, which was consistent with the different pathways in the microbe analysis. Thus, the HFD may induce obesity in mice through enhancing the levels of cholestanetriol, gingerol, and stevioside and decreasing the levels of isovaleric acid.

A total of 60 metabolites were identified as significantly different between the PS group and the OB group, including allopurinol, inosine, and L-ascorbic acid. Consistent with our study, allopurinol not only alleviates HFD-induced hypertension and proteinuria (El-Bassossy and Shaltout, 2015) but also ameliorates the HFD-induced hepatic steatosis through modulation of lipid metabolism, inflammation, and the ER stress pathway in mice (Cho et al., 2021). Niemann et al. (2022) demonstrated that inosine can combat obesity by regulating thermogenic fat, which is consistent with our findings. Lee et al. (2019) reported that ascorbic acid suppresses HFD-induced obesity and non-alcoholic fatty liver disease is activation of PPARα. KEGG analysis revealed that two amino acid metabolism and three energy metabolism-related pathways were enriched among the differential metabolites, aligning with the pathways identified in microbial analysis. Cluster analysis indicated that 74 metabolites may be associated with the PS-mediated alleviation of HFD-induced obesity in mice, including allopurinol (El-Bassossy and Shaltout, 2015), cholestanetriol (Zhu et al., 2021), gingerol (Saravanan et al., 2014), inosine (Niemann et al., 2022), L-ascorbic acid (Lee et al., 2019), and stevioside (Wang et al., 2012). Notably, the disrupted concentrations of inosine, L-ascorbic acid, stevioside, allopurinol, and gingerol in the OB group were restored by PS treatment. Furthermore, these 74 metabolites were enriched in two amino acid metabolism and three energy metabolism-related pathways, consistent with the microbial analysis. Collectively, PS might alleviate obesity in mice by restoring serum concentrations of inosine, L-ascorbic acid, stevioside, allopurinol, and gingerol and by regulating amino acid and energy metabolism-related pathways.

However, there were some limitations to our study. For instance, due to the short experimental period, the long-term effect might not be observed; the PS extraction method might have a possible influence on its active ingredients; the number of individuals in each group was small, and the PS administration groups with different doses were absent. We will improve these problems through further study.

## 5 Conclusion

In this study, an HFD was used to successfully establish a mice obesity model. Oral administration of PS significantly alleviated obesity, as evidenced by reductions in TG, TC, LDL-C, and HDL-C levels, as well as decreased adipocyte size. 16S rRNA sequencing analysis revealed that PS modulated the abundance of 17 microbial taxa associated with obesity mitigation, including *Alistipes, Lachnoclostridium, Odoribacter*, *Prevotellaceae UCG-001*, *Rikenellaceae RC9-gut group*, and *norank_g Bacteroidales S24-7 group*. Metabolomic analysis identified serum metabolites such as L-ascorbic acid, stevioside, allopurinol, and gingerol, along with amino acid metabolism and energy metabolism-related pathways, as key mechanisms underlying the anti-obesity effects of PS. These findings provide novel theoretical and experimental support for the potential application of PS in obesity prevention and treatment.

## Data Availability

The datasets presented in this study can be found in online repositories. The names of the repository/repositories and accession number(s) can be found below: https://ngdc.cncb.ac.cn/, CRA021056.
